# Nuclear overexpression of metastasis-associated protein 1 correlates significantly with poor survival in nasopharyngeal carcinoma

**DOI:** 10.1186/1479-5876-10-78

**Published:** 2012-04-26

**Authors:** Wen-Fei Li, Na Liu, Rui-Xue Cui, Qing-Mei He, Mo Chen, Ning Jiang, Ying Sun, Jing Zeng, Li-Zhi Liu, Jun Ma

**Affiliations:** 1 Department of Radiation Oncology, State Key Laboratory of Oncology in South China, Sun Yat-Sen University Cancer Center, 651 Dongfeng Road East, Guangzhou 510060, People’s Republic of China; 2 Department of Pathology, State Key Laboratory of Oncology in South China, Sun Yat-Sen University Cancer Center, 651 Dongfeng Road East, Guangzhou, 510060, People’s Republic of China; 3 Imaging Diagnosis and Interventional Center, State Key Laboratory of Oncology in South China, Sun Yat-Sen University Cancer Center, 651 Dongfeng Road East, Guangzhou, 510060, People’s Republic of China

**Keywords:** Nasopharyngeal carcinoma, Biomarker, MTA1, Prognosis

## Abstract

**Background:**

Metastasis-associated protein 1 (MTA1) has been associated with poor prognosis in several malignant carcinomas. The purpose of this study was to investigate the expression and prognostic value of MTA1 in nasopharyngeal carcinoma (NPC).

**Methods:**

MTA1 expression was assessed using immunohistochemistry in paraffin-embedded tumor specimens from 208 untreated NPC patients. Cox regression analysis was used to calculate the hazard ratio (HR), 95% confidence interval (CI) and identify independent prognostic factors, and recursive partitioning analysis was used to create a decision tree.

**Results:**

Nuclear overexpression of MTA1 was observed in 48.6% (101/208) of the NPC tissues. Nuclear overexpression of MTA1 correlated positively with N classification (*P* = 0.02), clinical stage (*P* = 0.04), distant metastasis (*P* < 0.01) and death (*P* = 0.01). Additionally, nuclear overexpression of MTA1 correlated significantly with poorer distant metastasis-free survival (DMFS; *P* <0.01) and poorer overall survival (OS; *P* < 0.01). MTA1 had prognostic significance in NPC patients with stage II disease, but not stage III or IV disease. Multivariate analysis demonstrated that nuclear overexpression of MTA1 was independently associated with poorer DMFS (HR, 2.05; 95% CI, 1.13–3.72; *P* = 0.02) and poorer OS (HR, 1.98; 95% CI, 1.09–3.59; *P* = 0.03). Using recursive partitioning analysis, the NPC patients could be classified with a low, intermediate or high risk of distant metastasis and death, on the basis of clinical stage, age and MTA1 expression.

**Conclusion:**

The results of this study suggest that nuclear overexpression of MTA1 correlates significantly with poorer DMFS and poorer OS in NPC. MTA1 has potential as a novel prognostic biomarker in NPC.

## Background

Nasopharyngeal carcinoma (NPC) is a unique type of head and neck malignancy with an extremely unbalanced endemic distribution. According to the International Agency for Research on Cancer, there were an estimated 84,400 incident cases of NPC and 51,600 deaths due to NPC in 2008, with 40% of these occurring in Chinese individuals [1]. Due to anatomic constraints and the high radiosensitivity, NPC is mainly treated with radiotherapy. Although recent advances in diagnosis and therapy have significantly improved treatment outcomes, distant metastasis still occurs in 20–30% of patients and becomes the major cause of death in NPC [2].

Currently, prediction of the risk of distant metastasis in NPC is still reliant on the TNM staging system; however, varying outcomes are observed in patients with the same stage of disease who receive similar therapies, indicating that some NPC patients may be benefit from more aggressive treatments, while others could potentially be spared the toxicity of unnecessary chemotherapy. Although several biomarkers have been associated with prognosis in NPC, prognostic prediction in NPC is still dismal [3]. Hence, it is of great clinical value to identify novel biomarkers which could be utilized as effective prognostic predictors or possible therapeutic targets, in order to optimize the treatment of patients with NPC.

The metastasis**-**associated protein (MTA) family is a group of structurally related proteins encoded by the same or different genes, including MTA1, MTA1s, MTA-ZG29p, MTA2, MTA3, and MTA3L [4,5]. MTA proteins physically interact with histone deacetylase 1 (HDAC1) and HDAC2 to form the nucleosome remodeling and histone deacetylation (NuRD) protein complex, which is involved in chromosome remodeling, histone deacetylation and transcriptional regulation [6,7]. As a major member of the MTA family, the MTA1 gene was first identified by differential screening of cDNA libraries constructed from highly metastatic and nonmetastatic mammary adenocarcinoma cell lines [8,9]. Experimental studies have shown that MTA1 may play an important role in carcinogenesis and cancer progression, including malignant transformation, angiogenesis, invasion and metastasis, by interacting with various cell signaling pathways [4,5,10]. MTA1 is upregulated in diverse human malignancies, including carcinoma of the stomach, esophagus, prostate, breast, liver, oral cavity, lung and colorectum, and is associated with tumor progression, lymph node metastasis and poor prognosis [11-20]; however, little is known about the expression level and prognostic value of MTA1 in NPC.

Therefore, in this report, we studied the expression pattern of MTA1 in tumor samples from NPC patients, and analyzed the association between MTA1 expression and the clinicopathological characteristics of NPC. In addition, we evaluated the prognostic value of MTA1, in order to develop more personalized therapy for NPC patients.

## Methods

### Patient and tissue specimens

A total of 208 pretreatment paraffin-embedded NPC specimens were collected from the archives of the Department of Pathology of the Cancer Center of Sun Yat-sen University between January 2004 and February 2006. The cases were selected on the basis of the following criteria: histologically proven NPC with available biopsy specimens, newly diagnosed and non-distant metastatic NPC; no treatment history or other malignant disease; Karnofsky score ≥70; received radiotherapy at our Cancer Center and follow-up regularly. The study was approved by the Institutional Research Ethics Committee and written informed consent was obtained from each participant. All medical records were reviewed retrospectively and all patients were restaged according to the 7th edition of the UICC/AJCC system [21]. All 208 patients were pathologically diagnosed with World Health Organization type II or III NPC, and the clinicopathological characteristics of the patients are summarized in Table 1.

**Table 1:** Correlation between the nuclear expression levels of MTA1 and the clinicopathological characteristics of nasopharyngeal carcinoma patients

**Characteristic**	**No. of patients**	**MTA1 Nuclear Expression**		**Chi-square test**
		**Low, n(%)**	**High, n(%)**	***P*value**
**Sex**				0.93
Male	155	80 (51.6%)	75 (48.4%)	
Female	53	27 (50.9%)	26 (49.1%)	
**Age(y)**				0.86
≤ 50	129	67 (51.9%)	62 (48.1%)	
> 50	79	40 (50.6%)	39 (49.4%)	
**T classification**				0.07
T1-T2	104	60 (57.7%)	44 (42.3%)	
T3-T4	104	47 (45.2%)	57 (54.8%)	
**N classification**				0.02
N0-N1	130	75 (57.7%)	55 (42.3%)	
N2-N3	78	32 (41.0%)	46 (59.0%)	
**Clinical stage**				0.04
I-II	70	43 (61.4%)	27 (38.6%)	
III-IV	138	64 (46.4%)	74 (53.6%)	
**Locoregional failure**				0.61
No	185	94 (50.8%)	91 (49.2%)	
Yes	23	13 (56.5%)	10 (43.5%)	
**Distant metastasis**				<0.01
No	159	90 (56.6%)	69 (43.4%)	
Yes	49	17 (34.7%)	32 (65.3%)	
**Death**				0.01
No	160	90 (56.2%)	70 (43.8%)	
Yes	48	17 (35.4%)	31 (64.6%)	

### Treatment and follow-up

All patients received definitive radiotherapy: 160/208 (76.9%) patients were treated with two-dimensional radiotherapy (2D-RT) and 48/208 (23.1%) patients were treated with intensity-modulated radiotherapy (IMRT). Details of the radiation therapy techniques applied at the Cancer Center of Sun Yat-Sen University have been reported previously [2]. During the study period, the institutional guidelines recommended no chemotherapy for stage I to IIa patients, concurrent chemoradiotherapy for stage IIb patients, and concurrent chemoradiotherapy with or without neoadjuvant/adjuvant chemotherapy for stage III to IVa-b patients, as defined by the 6th edition of the UICC/AJCC staging system for NPC. Concurrent chemotherapy consisted of cisplatin administered on weeks 1, 4 and 7 of radiotherapy, or weekly. Neoadjuvant and adjuvant chemotherapy consisted of cisplatin with 5-fluorouracil or taxanes every three weeks for three cycles. Overall, 62.5% (40/64) of the stage IIB patients and 84.1% (116/138) of the stage III-IV patients received chemotherapy.

The median follow-up period for the entire cohort was 62.4 months (range, 5.2–79.9 months). The 5-year overall survival (OS), distant metastasis-free survival (DMFS) and locoregional relapse-free survival rates were 78.4%, 76.7% and 87.4%, respectively.

### Immunohistochemistry (IHC)

Expression of MTA1 was examined using IHC in tissue sections from 208 cases of human NPC. The sections were deparaffinized in xylene, rehydrated through graded alcohols, immersed in 3% hydrogen peroxide for 30 min to quench endogenous peroxidase activity, and microwaved in EDTA antigen retrieval buffer. Nonspecific binding was blocked with 1% bovine serum albumin at room temperature for 20 min, then the sections were incubated with anti-MTA1 mouse monoclonal antibody (1:500 dilution; Abcam, Cambridge, UK) overnight at 4°C in a humidified chamber. Negative controls were performed by replacing the primary antibody with normal goat serum. After washing, the tissue sections were incubated with biotinylated anti-mouse secondary antibody for 30 min at room temperature followed by streptavidin-horseradish peroxidase complex for 20 min, stained with 3, 3-diaminobenzidine (DAB), counterstained with hematoxylin, dehydrated and mounted.

### IHC scoring

The degree of immunohistochemical staining was evaluated independently by two clinical pathologists who were blinded to the clinicopathological data, and any differences were discussed until consensus was reached. MTA1 nuclear immunoreactivity was scored using a semiquantitative scoring system incorporating the proportion of positively stained tumor cells (0, ≤5%; 1, 6–25%; 2, 26–50%; 3, 51–75%; 4, ≥76%) and the intensity of staining (0, no staining; 1, light yellow weak staining; 2, yellow brown moderate staining; 3, brown strong staining) to result in a score of 0, 1, 2, 3, 4, 6, 8, 9 or 12. The cutoff value for high- and low-expression was selected on the basis of a measure of heterogeneity using the log-rank test statistical analysis with respect to overall survival. The optimal cutoff value was defined as 4: tumors with scores ≤ 4 were defined as low MTA1 expression, while tumors with scores > 4 were designated as high MTA1 expression.

### Statistical analysis

The *χ*^2^-test was used to analyze the correlation between MTA1 expression and clinicopathological features. The actuarial rates were calculated using the Kaplan-Meier method [22] and differences were compared using the log-rank test. Multivariate analysis using the Cox proportional hazards model [23] was performed to calculate the hazard ratio (HR), 95% confidence interval (CI) and to test independent significance by backward elimination of insignificant variables. Covariates included host factors (i.e., sex, age), therapeutic intervention (i.e., radiotherapy technique, chemotherapy) and tumor factors (i.e., T classification, N classification, clinical stage). These analyses were performed using Statistical Package for the Social Sciences (SPSS) v13.0 (Chicago, IL, USA); two-tailed *P*-values < 0.05 were considered statistically significant. Recursive partitioning analysis was performed to construct prognostic models using the significant variables from the multivariate analysis. R software (R version 2.14.1; rpart package version 3.1–51, http://www.r-project.org/) was used to create a decision tree with split criteria, based on the log-rank test.

## Results

### Expression of MTA1 in NPC

**Figure 1:**
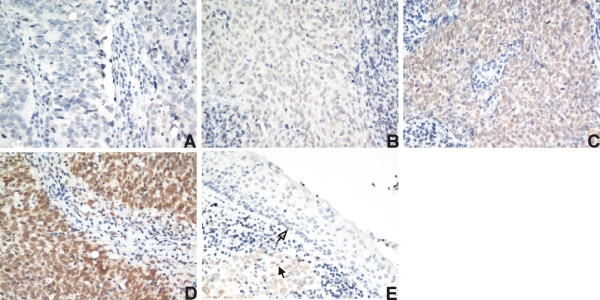
**Immunohistochemical analysis of MTA1 protein expression in nasopharyngeal carcinoma tissues.** MTA1 protein expression was mainly localized within the nucleus. (A) Negative staining (400×); (B) weak staining: light yellow (400×); (C) moderate staining: yellow brown (400×); (D) strong staining: brown (400×); (E) MTA1 protein expression was upregulated in the nasopharyngeal carcinoma tissue (solid arrow) compared to the adjacent noncancerous epithelial cells (hollow arrow).

Representative images of MTA1 immunohistochemical staining in NPC tissues are shown in Figure 1A-D. Positive MTA1 expression was observed in the nuclei of NPC cells. High nuclear expression of MTA1 was detected in 48.6% (101/208) of the NPC tissues, while weak or no nuclear MTA1 staining was observed in the adjacent noncancerous epithelial cells. As shown in Figure 1E, MTA1 was overexpressed in the nuclei of NPC cells compared to the adjacent normal epithelial cells in the same section.

### Correlation between MTA1 expression and the clinicopathological features of NPC

Table 1 shows the relationship between MTA1 protein expression and the clinicopathological characteristics of NPC patients. Nuclear overexpression of MTA1 correlated positively with N classification (*P* = 0.02), clinical stage (*P* = 0.04), distant metastasis (*P* < 0.01) and death (*P* = 0.01). However, there was no significant correlation between MTA1 expression and other clinicopathological characteristics, such as age, gender, T classification or locoregional failure (*P* > 0.05).

#### Univariate analysis

**Figure 2:**
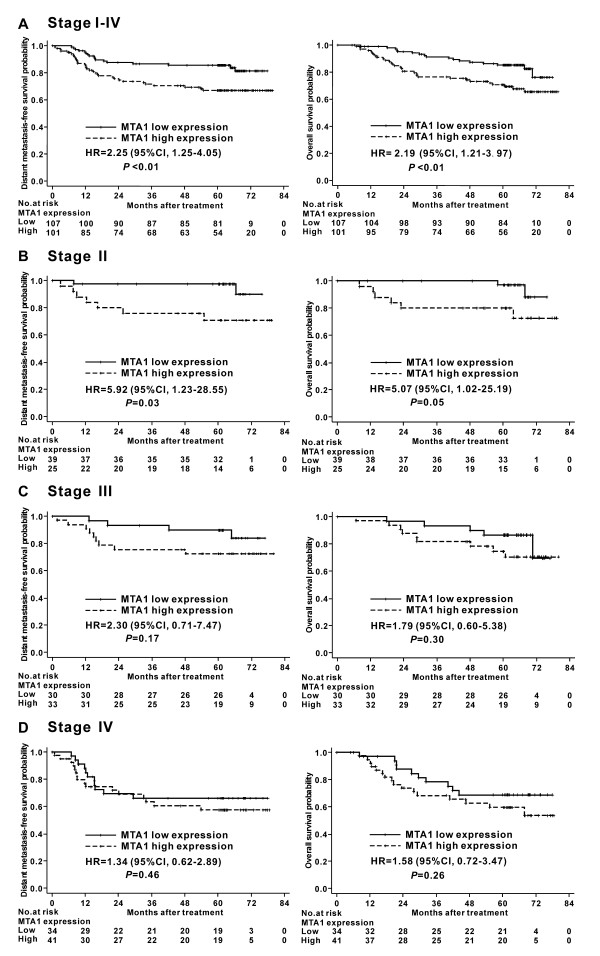
**Kaplan-Meier distant metastasis-free survival and overall survival curves for nasopharyngeal carcinoma patients stratified by MTA1 expression level and clinical stage.** (A) Stage I-IV patients (n = 208); (B) Stage II patients (n = 64); (C) Stage III patients (n = 63); (D) Stage IV patients (n = 75). HR: hazard ratio; CI: confidence interval.

Gender, radiation technique, chemotherapy and T classification had no significant impact on DMFS or OS in univariate analysis (*P* > 0.05). In contrast, age, N classification, clinical stage and MTA1 expression could significantly predict DMFS and OS (*P* < 0.05; Table 2). Kaplan-Meier analysis revealed that nuclear overexpression of MTA1 correlated significantly with poorer DMFS (HR, 2.25; 95% CI, 1.25–4.05; *P* < 0.01) and poorer OS (HR, 2.19; 95% CI, 1.21–3.97; *P* < 0.01; Figure 2A).

**Table 2:** Univariate analysis of the significance of different prognostic variables in nasopharyngeal carcinoma

**Factors**	**DMFS**			**OS**		
	***P*value**	**HR**	**95% CI**	***P*value**	**HR**	**95% CI**
**Age** (>50 yr vs. ≤50 yr)	0.02	1.90	1.09–3.35	<0.01	2.15	1.22–3.81
**Sex** (female vs. male)	0.82	0.93	1.48–1.78	0.66	0.86	0.44–1.69
**Chemotherapy** (yes vs. no)	0.14	1.78	0.83–3.79	0.26	1.52	0.73–3.13
**Radiotherapy** (IMRT vs.2D-RT)	0.99	1.00	0.80–1.25	0.93	1.01	0.81–1.26
**T stage** (T_3–4_ vs. T_1–2_)	0.13	1.55	0.88–2.74	0.06	1.77	0.99–3.18
**N stage** (N_2–3_ vs. N_0–1_)	0.02	1.98	1.13–3.47	0.01	2.06	1.17–3.63
**Clinical stage** (III-IV vs. I-II)	0.01	2.52	1.22–5.19	<0.01	2.78	1.30–5.95
**MTA1 expression** (high vs. low)	<0.01	2.25	1.25–4.05	<0.01	2.19	1.21–3.97

Abbreviation: *DMFS* distant metastasis free-survival; *OS* overall survival; *IMRT* intensity-modulated radiotherapy; *2D-RT* two-dimensional radiotherapy.

We further analyzed the prognostic value of MTA1 in subgroups of NPC patients stratified according to clinical stage. As only 6 patients had stage I disease, the stratified analysis was performed in stage II-IV patients. In the stage II subgroup, patients with nuclear overexpression of MTA1 had significantly poorer DMFS (HR, 5.92; 95% CI, 1.23–28.55; *P* = 0.03) and poorer OS (HR, 5.07; 95% CI, 1.02–25.19; *P* = 0.05) than patients with lower levels of MTA1 expression (Figure 2B). However, the OS and DMFS of stage III or IV patients with low and high MTA1 expression were not significantly different (Figure 2C-D).

#### Multivariate analysis

Multivariate analysis, which included age (>50 yr vs. ≤50 yr), sex (female vs. male), radiotherapy (IMRT vs. 2D-RT), chemotherapy (yes vs. no), clinical stage (stage III-IV vs. I-II) and the MTA1 protein expression level (high vs. low), was performed to identify independent prognostic factors. The expression level of MTA1 was an independent prognosticator for DMFS (HR, 2.05; 95% CI, 1.13–3.72; *P* = 0.02) and OS (HR, 1.98; 95% CI, 1.09–3.59; *P* = 0.03). From the other variables, age and clinical stage were also found to be independent prognostic factors for DMFS and OS (Table 3).

**Table 3:** Multivariate analysis of the significance of different prognostic variables in nasopharyngeal carcinoma

**Factors**	**DMFS**			**OS**		
	***P*value**	**HR**	**95% CI**	***P*value**	**HR**	**95% CI**
**Age** (>50y vs. ≤50y)	0.02	1.91	1.09–3.34	0.01	2.11	1.19–3.74
**Sex** (female vs. male)	0.88	1.05	0.54–2.05	0.99	1.00	0.50–2.01
**Chemotherapy** (yes vs. no)	0.39	1.42	0.64–3.11	0.62	1.21	0.57–2.58
**Radiotherapy** (IMRT vs.2D-RT)	0.65	0.95	0.76–1.19	0.67	0.95	0.76–1.20
**Clinical stage** (III-IV vs. I-II)	0.03	2.21	1.06–4.59	0.02	2.44	1.13–5.25
**MTA1 expression** (high vs. low)	0.02	2.05	1.13–3.72	0.03	1.98	1.09–3.59

Abbreviation: *DMFS* distant metastasis free-survival; *OS* overall survival; *IMRT* intensity-modulated radiotherapy; *2D-RT* two-dimensional radiotherapy.

#### Recursive partitioning analysis

**Figure 3:**
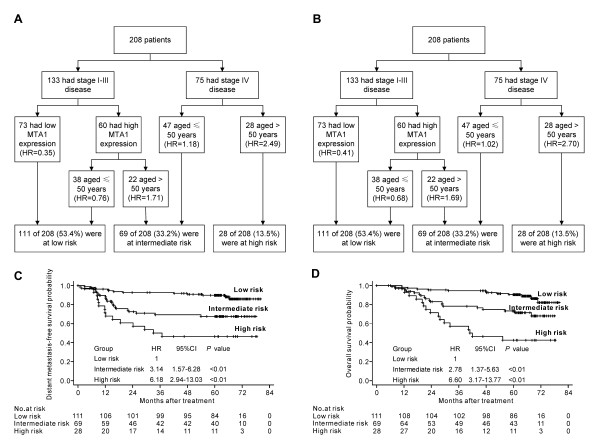
**(A) Recursive partitioning analysis of distant metastasis-free survival in all nasopharyngeal carcinoma (NPC) patients; (B) recursive partitioning analysis of overall survival in all NPC patients; (C) Kaplan-Meier distant metastasis-free survival curves in the classified patients; (D) Kaplan-Meier overall survival curves in the classified patients.** HR: hazard ratio; CI: confidence interval.

Recursive partitioning analysis was performed to construct a decision tree, using the significant independent prognostic factors for DMFS and OS including age, clinical stage and MTA1 expression. The same decision tree was produced using distant failure and death as endpoints (Figure 3A-B). Based on the HR calculated in each terminal node, we classified the patients into low, intermediate and high risk groups, with 5-year DMFS and OS rates of 89.8% and 90.6%, 67.6% and 73.3%, 46.4% and 42.6%, respectively. Significant differences were observed between the groups (Figure 3C-D). Compared with the low risk group (HR = 1), the intermediate risk group and the high risk group both had significantly higher HRs for DMFS and OS (*P* < 0.01).

## Discussion

The recognition that cancers of the same clinical stage have divergent prognoses and respond differently to the same therapeutic intervention has promoted the study of prognostic markers, which is particularly important for the development of personalized therapy. In the present study, we observed that the nuclear expression levels of MTA1 correlated significantly with the clinical stage and survival of NPC patients. In addition, MTA1 was identified as an independent prognosticator in multivariate analysis, indicating that MTA1 has potential as a novel prognostic biomarker to guide clinical practice and research on NPC.

The majority of NPC deaths are attributed to tumor metastases, rather than local failure. As a metastasis-associated protein, overexpression of MTA1 has been demonstrated in a range of malignant tumors and is strongly associated with cancer cell invasion and migration. Moreover, overexpression of MTA1 has been clinically linked to advanced clinical stage, lymph node metastasis and poor prognosis [11-20]. Recently, Deng et al. used in situ hybridization to detect the expression of *MTA1* mRNA in NPC. The positive rate of *MTA1* expression was significantly higher in NPC and metastatic lymph node tissues than chronic nasopharyngitis tissues, and the expression level of *MTA1* mRNA correlated positively with lymph node metastasis, tumor recurrence and death; however, the prognostic significance of MTA1 was not investigated [24].

In this study, we examined the expression of MTA1 in paraffin-embedded NPC biopsies using immunohistochemical staining. We observed that 48.6% of the specimens expressed high levels of MTA1 in the tumor cell nuclei, while MTA1 was absent or expressed weakly in the nuclei of the adjacent noncancerous epithelial cells. We also showed that nuclear overexpression of MTA1 correlated significantly with advanced N classification and clinical stage as well as distant metastasis and death, but not with T classification or locoregional failure. These findings strongly suggest that MTA1 may play an important role in the development and progression of NPC.

Furthermore, nuclear overexpression of MTA1 was associated with poorer DMFS and poorer OS in both univariate and multivariate analysis, suggesting that nuclear overexpression of MTA1 is an unfavorable prognostic factor in NPC. It is noteworthy that the prognostic value of MTA1 was significant in stage II NPC patients in the stratified analysis, but not stage III or IV patients. In order to combine MTA1 with known robust clinical prognostic factors, we established a recursive partitioning tree consisting of MTA1, clinical stage and age, which were independent prognostic factors for DMFS and OS in multivariate analysis. The study population could be segregated into three distinct prognostic groups: MTA1 had specific prognostic value in patients with stage I-III disease, while age was evaluated as the most important prognosticator in the stage IV subgroup.

Our results indicate that the prognostic value of MTA1 is most significant in early-stage NPC patients, especially those with stage II disease, and similar findings have also been reported in other types of cancer [18,20]. According to the National Comprehensive Cancer Network guidelines, NPC patients with stage II disease should receive concurrent chemoradiotherapy followed by adjuvant chemotherapy. However, excellent survival rates for stage II NPC patients treated with concurrent chemoradiotherapy alone have been reported recently [25], indicating that some patients could possibly be spared from the toxicity and cost of unnecessary adjuvant chemotherapy. The results of this study suggest that MTA1 may provide an effective prognostic index to identify individuals with a poor prognosis, and help to stratify the need for adjuvant chemotherapy in stage II NPC patients.

Further investigation of the function and mechanism of action of MTA1 may provide additional targets and strategies for the treatment of NPC. To date, MTA1 has been demonstrated to be involved in multiple signaling pathways, which contribute to several aspects of the metastatic phenotype by modifying the acetylation status of crucial target genes [4,5,10]. Yoo et al. reported that MTA1 induced the deacetylation and enhanced the stability of hypoxia-inducible factor-1α (HIF-1α) by recruiting HDAC1 in breast cancer cells, which indicates that a close connection may exist between MTA1-associated metastasis and HIF-1α-induced tumor angiogenesis [26]. Kai et al. identified MTA1 as a component of a bone metastasis signature in prostate cancer, and MTA1-knockdown cells expressed lower levels of vascular endothelial growth factor (VEGF) and displayed decreased angiogenicity both in vitro and in vivo [27], suggesting that MTA1 upregulates VEGF expression, in agreement with Du et al. [19]. Correlations between the expression of MTA1 and matrix metalloproteinase 9 [28], E-cadherin [29] and the cellular cytoskeleton components [30] have also been reported by different researchers.

The mechanisms leading to the upregulation of MTA1 protein expression in human cancers are not known. Zhang et al. reported that MTA1 was an essential downstream effector of the c-MYC oncoprotein and regulated the epithelial-to-mesenchymal transition (EMT) process [31]. Recently, Lee et al. reported that a *MTA1* IVS4-81 G/A single nucleotide polymorphism (SNP) and *VEGF* +12916C SNP were associated with overexpression of MTA1 in hepatocellular carcinoma tissues [32]. Our study suggests that MTA1 plays an important role in the development and progression of NPC; however, further investigation is required to clarify its mechanism of action and determine the precise signaling pathways by which MTA1 regulates metastasis in NPC.

## Conclusions

In summary, this study revealed the expression pattern of MTA1 in NPC, and demonstrated that high levels of nuclear MTA1 expression correlated significantly with poorer DMFS and OS in NPC, especially in patients with stage II disease. Furthermore, incorporation of MTA1 with age and clinical stage enabled the classification of NPC patients into groups with a low, intermediate, or high risk of distant metastasis and death using recursive partitioning analysis. Our results suggest that upregulation of MTA1 may be important for the acquisition of a poor prognostic phenotype in NPC. However, these findings need to be replicated in a different patient population, and further studies are needed to clarify the mechanism by which MTA1 is involved in the development and progression of NPC.

## Misc

Wen-Fei Li and Na Liu contributed equally to this article

## Competing interests

The authors indicate no actual or potential competing interests exist.

## Authors’ contributions

JM, YS and WFL were responsible for the study design. WFL, QMH and RXC carried out the immunohistochemical staining work. WFL, NL, MC, JZ, NJ and LZL participated in the data collection and analysis. WFL and NL drafted the manuscript and participated in the data interpretation. All authors read and approved the final manuscript.
